# Learning about Haemophilia

**Published:** 1986-12

**Authors:** Carol Kendrick

**Affiliations:** Medical Student, University of Bristol Medical School

## Abstract

In order to learn about Haemophilia from the point of view of the sufferers, I interviewed six severely affected haemophiliacs, discussing with them their past experience, current status and treatment. I found them to be an optimistic and independent group who, despite their memories of a painful childhood and their current disablement, contrived to live in the present with gusto and vigour. They were very knowledgeable about Haemophilia and were happy to teach me, thus providing me with a valuable body of knowledge and ideas on the subject.


### Learning About Haemophilia: An Alternative Source of Information

**Carol Kendrick, BSc (Lond.)**
Medical Student, University of Bristol Medical School

### ABSTRACT

In order to learn about Haemophilia from the point of view of the sufferers, I interviewed six severely affected haemophiliacs, discussing with them their past experience, current status and treatment. I found them to be an optimistic and independent group who, despite their memories of a painful childhood and their current disablement, contrived to live in the present with gusto and vigour. They were very knowledgeable about Haemophilia and were happy to teach me, thus providing me with a valuable body of knowledge and ideas on the subject.

### INTRODUCTION

As a 4th year student, having followed a very conventional medical course consisting of lectures, ward rounds and textbook consultation, I had the chance to try out a new form of learning in the Haematology Department. For my project in the Pathology course I set out to learn about Haemophilia by talking with a number of men who suffer from the disease. These men had made a lifelong study of the subject from necessity, and were only too pleased to share their specialist knowledge with me. By talking at length with six severely affected men I was able to learn about various aspects of Haemophilia in a very memorable fashion. Since the men, aged 23–57 years, had all grown up in an era before the widespread availability of replacement blood products for therapy, I also obtained a fascinating picture of how the course of a disease can be affected by the introduction of effective treatment. I would like to present an account of Haemophilia as provided by the sufferers themselves.

### METHODS

On the advice of my supervisor in the Haematology Department I wrote to six patients registered at the Bristol Haemophilia Centre explaining the aim of the project and requesting an interview. Each of the six men agreed, and the interviews took place either in the men's own homes or in the department at the hospital. Further individual details about the men interviewed are shown in *Table 1*. The interviews lasted from one to three and a half hours, were informally structured, and covered the following aspects of the men's lives: dependence; disability; knowledge about Haemophilia; other people's attitudes; and childhood memories.

**Table 1**

**Personal Details of the Six Men Interviewed**

| *Individual* | *Age* | *Occupation* | *Civil status* | *Domestic status* |
|---|---|---|---|---|
| A | 23 | Post-graduate student | Single | Lives alone |
| B | 27 | Accountant | Married | Lives with wife |
| C | 31 | Civil Servant | Single | Lives with parents |
| D | 42 | Unemployed | Married | Lives with wife |
| E | 46 | Civil Servant | Single | Lives with parents |
| F | 57 | Theology Student | Divorced | Lives with companion |

### DISABILITY

As a result of haemarthroses experienced during early childhood and teenage years, all of the men were, in strictly medical terms, disabled. Their X-rays showed substantial evidence of joint disease. On first meeting them, their lack of mobility was obvious. With the exception of *A*, the youngest man in the group, they all used a walking stick, crutches or wheelchair to get around. This disabled state was acknowledged by the receipt of the DHSS mobility allowance by all but one (*A*) of the men. In talking to these men about their lives, however, it was clear that they had all managed to compensate for this physical impairment in a most remarkable manner. Five out of the six men were either in full time employment or were studying full time. Between them they boasted a number of absorbing and in some cases energetic leisure pursuits indicating that they were, on the whole, leading very full and satisfying lives. Each of the men emphasised the importance of items such as a fridge, car and telephone (DHSS allowances ensure that all haemophiliacs in the UK are able to obtain these facilities) in overcoming their disabilities. In addition, it was apparent that their difficulties as a result of physical impairment were greatly diminished by the help they received from family and friends. For example, *B*'s wife would drive to the hospital to collect supplies and generally boost his morale if he was stuck at home with a bleed.

*F* sums up by saying:

'A haemophiliac is an ordinary person. I mean he is disabled, but he's ordinary. He just needs an able bodied person to rely on.'

### DEPENDENCE

The question of dependence on other people was a very relevant area for these men. They were, of course, all obliged to be dependent on the staff at the Haemophilia Centre from whom they obtained their very necessary blood products. Five of them were on home therapy and were therefore independent to that extent. With the exception of *A* they were all living with someone who was prepared to support and assist them. *F*, whose religious views led him to believe that he was entitled to be cared for by the community, was quite content to be dependent on a companion at home. *C*, the one man not on home treatment, showed an altogether more dependent attitude to the medical profession. He was docile, compliant, and made numerous attempts to please the doctors and nurses at the Centre. However, his leisure activities, which included gliding and travelling round Europe as a football supporter, displayed a more independent streak. *D*, an unemployed man, had developed his artistic skills. He was financially as well as psychologically dependent on his wife and stated categorically that he would never want anyone (meaning either children or pets) dependent on him. His independence came in the form of the theories he held about Haemophilia, as well as his art work.

### KNOWLEDGE AND THEORIES ABOUT HAEMOPHILIA

All of the men I talked with were extremely knowledgeable about their disease, and were able to teach me a great deal about clotting factors, blood products, genetics and the treatment of Haemophilia. Their practical ability to ascertain when they had a bleed, as well as their knowledge of how and when to treat, was evident. All except one (*C*) had mastered the technique of intravenous administation and felt competent to deal with all but the most serious of bleeds themselves. They did in some cases, however, hold rather idiosyncratic views. For example, *D*, referred to earlier, stated 'I use 3000 units [as opposed to the 1000 units recommended]. You have to hit it [a bleed] hard at the beginning. I've got to know what suits me, and mostly I'm right. I expect the doctors to listen to me because of my experience.'

### OTHER PEOPLE'S ATTITUDES

All of the group felt that the public was very ill-informed about Haemophilia and its treatment nowadays. It was thought that there was a widespread fear of the disease because of the idea of uncontrolled bleeding, and more recently of AIDS. *E*: 'People don't know about haemophilia. I don't tell them, they only get worried that you will bleed to death if you cut yourself shaving.'

The question of prejudice against haemophiliacs also came up in relation to employment. For example, *C* had to struggle, even thirteen years ago when the job situation was less bleak, to get accepted as a Civil Servant. He was forced to serve a three-year probation period as well as producing various letters from the hospital in order to gain employment.

The men were on the whole reluctant to tell even their friends about their haemophilia unless forced to. *D*: 'I try to avoid telling anyone if at all possible. Your boss has to know, otherwise you may end up getting the sack; on the other hand, if you do tell him you've less chance of getting the job. It seems you just can't win.'

This general lack of information about haemophilia was felt to extend to hospital staff (outside the Haematology Department). The men in many cases felt misunderstood when they came in contact with other doctors and fled rapidly back to the Haemophilia Centre staff at the first possible opportunity. *B* explained how he had once arrived in Casualty on a Saturday night with a bleed he could not control adequately at home. 'First they thought I was a drug addict because of the syringe I had brought along. Then, when they finally did believe that I was a haemophiliac, that sent them into a flat spin.'

### CHILDHOOD

When they described their childhood experiences, it was apparent that all of the men had led very restricted lives as schoolboys. There had been no question of any sporting activities or travel for any of them.

*F*, sent to a special school for the physically and mentally handicapped, managed to escape from there by learning to play the piano and subsequently winning a scholarship to the Royal Academy of Music. 'You see, playing the piano was the only thing I *could* do as a child,' he said.

*A* missed a great deal of school as a child because of his numerous hospital confinements with bleeds. 'Mind you,' he said, 'I never really fitted in at school, the headmaster took me on as a sort of experiment. All the other boys were told that I was fragile and they mustn't touch me, as a result they hardly even spoke to me. I was pretty lonely.'

*D* and his brother, also a haemophiliac, never went to school at all. They had home tuition for one hour per week, and that was it. (*D* is the one man in the group who is now unemployed).

Some of the men reported that treatments of an experimental nature had been tried on them. *C*, for example, was put on a diet of peanut butter sandwiches for six months. *D* had his mother's blood smeared on to the surface of his own bleeds.

The main memory of childhood for all the men, however, was that of the pain they had suffered, and of resting their bleeding, swollen joints endlessly in bed because of that pain, which was variously described as 'diabolical' and 'sheer bloody hell'. *B* said 'Some days I just thought it would be easier to die.' *D* stated more philosophically, 'You just bled in them days. If it didn't kill you, you were doing pretty well.'

### CONCLUSIONS

I consider myself fortunate to have had the opportunity to learn about haemophilia from the patients themselves. In sharing their knowledge and thoughts on the subject so generously they provided me with a unique insight into what it means to be living with a chronic disease such as haemophilia. I was impressed with the fullness of their lives and by the way in which they had come to terms with their own disabilities. It was intriguing to discover that each man considered himself to be special amongst the haemophiliacs of his generation in terms of his achievements. The men, although showing great independence in some aspects of their lives, were emphatically acknowledging the vital support they received from family, friends and familiar staff at the Haemophilia Centre. The misunderstandings and prejudices that still surround haemophiliacs, even from within the medical profession, provides cause for concern. The painful memories of childhood, as recounted by these men, which thankfully will not be reproduced in the latest generation of haemophiliacs, enabled me to comprehend more fully the nature of the disease process.

I would certainly recommend this form of learning to other aspiring doctors.

### ACKNOWLEDGEMENTS

I would like to thank Dr Helena M Daly, who supervised this project, introduced me to the patients, and enthusiastically supported me throughout. I am most grateful to the six men who participated in the study for sharing their knowledge and ideas with me most generously. Finally, I would like to thank Dr G L Scott and Sister P A Pennock, who provided me with encouragement and helpful ideas.

